# Association of anticoagulant and antiplatelet therapy with acute cerebral infarction in patients presenting with isolated vertigo or dizziness: A retrospective cohort study

**DOI:** 10.1371/journal.pone.0350671

**Published:** 2026-06-11

**Authors:** Dongwook Kim, Sion Jo, Inha Hwang, Boyoung Park, Min Joung Kim

**Affiliations:** 1 Department of Medicine, Yonsei University College of Medicine, Seoul, Republic of Korea; 2 Department of Emergency Medicine, Veterans Health Service Medical Center‌‌, Seoul, Republic of Korea; 3 Veterans Medical Research Institute, Veterans Health Service Medical Center, Seoul, Republic of Korea; 4 Department of Medicine, College of Medicine, Hanyang University, Seoul, Republic of Korea; 5 Department of Emergency Medicine, Yonsei University College of Medicine, Seoul, Republic of Korea; Fondazione Policlinico Universitario Agostino Gemelli IRCCS, ITALY

## Abstract

**Background:**

Acute cerebral infarction (ACI) is a critical concern in patients presenting with isolated vertigo or dizziness (IVD). The use of antiplatelet or anticoagulant agents may influence the incidence of ACI in both protective and adverse directions, due to their preventive effects and associated comorbidities. This study aimed to determine the incidence of ACI among emergency department (ED) patients with IVD, stratified by the use of antiplatelet and anticoagulant medications. We also evaluated the association between antiplatelet and anticoagulant therapies and ACI incidence.

**Methods:**

This was a retrospective cohort study. ED patients with IVD over four consecutive years were identified. The main outcome was ACI, which was determined by brain magnetic resonance imaging with diffusion-weighted imaging (bMRI-DWI). The primary independent variable was the use of antiplatelet or anticoagulant medications, which was categorized into two models. In Model 1, patients were divided into three groups: a no-medication group (patients who did not take any antiplatelet or anticoagulant agents), an antiplatelet group (patients who took aspirin or clopidogrel but no anticoagulants), and an anticoagulant group (patients who took warfarin or non–vitamin K antagonist oral anticoagulants [NOACs], regardless of concurrent antiplatelet use). Model 2 provided a more detailed classification of medication use: no-medication group, aspirin-only group, clopidogrel-only group, aspirin and clopidogrel group, and anticoagulant group. In addition, based on whether comorbidities were adjusted for, the multiple logistic regression models were categorized into Model A (without adjustment for comorbidities) and Model B (with adjustment for comorbidities). A total of four multivariable logistic regression models were developed for analysis.

**Results:**

Among the 1,875 patients enrolled, 153 (8.2%) were diagnosed with ACI. The incidence of ACI was 6.7% (74/1,104) in the no-medication group, 8.8% (59/670) in the antiplatelet group, and 19.8% (20/101) in the anticoagulant group. In the more detailed classification, the incidence was 7.4% (28/377) in the aspirin-only group, 6.6% (10/152) in the clopidogrel-only group, and 14.9% (21/141) in the dual antiplatelet group (aspirin and clopidogrel). In all four multivariable models, the use of anticoagulants emerged as an independent predictor of ACI (adjusted odds ratio [AOR], 2.46; 95% confidence interval [CI], 1.20–5.08; p = 0.014 in Model 2B). The dual antiplatelet group was significantly associated with ACI in Model 2A (AOR, 1.74; 95% CI, 1.02–2.97; p = 0.044), but this association was no longer significant after adjusting for comorbidities in Model 2B (AOR, 1.34; 95% CI, 0.77–2.34; p = 0.297). The antiplatelet group, aspirin-only group, and clopidogrel-only group were not significantly associated with ACI occurrence in any model.

**Conclusion:**

Nearly 20% of patients in the anticoagulant group and 15% in the dual antiplatelet group (aspirin and clopidogrel) experienced ACI among ED patients with IVD. The use of anticoagulants was a significant predictor of ACI occurrence. The association between dual antiplatelet therapy and ACI lost statistical significance after adjusting for comorbidities. These two medication groups may serve as clinical proxies for elevated ACI risk in ED patients presenting with IVD.

## Introduction

Each year, approximately 7.5 million ambulatory care visits in the United States are attributed to dizziness [1]. Dizziness also accounts for approximately 4% of emergency department (ED) visits [2]. A wide range of conditions, from benign to life-threatening, can cause dizziness [3]. Coexisting symptoms, physical signs, and laboratory or radiologic findings assist clinicians in identifying the underlying cause. However, diagnosing patients with isolated vertigo or dizziness (IVD) is particularly challenging. Small acute cerebral infarction (ACI), especially those involving the cerebellum, may present without other focal neurologic deficits [4]. Because neurologic deficits are absent, clinicians may easily overlook the possibility of ACI in patients with IVD. These features of IVD complicate the decision to perform brain magnetic resonance imaging (bMRI), which is costly.

Previous studies have reported ACI incidence among IVD patients ranging from 0.7% to 17% [5–10]. The author’s previous study found an ACI incidence of 2.8% (13/468) among IVD patients presenting to a single urban university hospital in South Korea [11]. Therefore, sharing information with patients and caregivers regarding the potential incidence of ACI in IVD cases is an important step toward appropriate MRI utilization.

Recently, several clinical assessment protocols, including the HINTS Examination [12], the STANDING Algorithm [13], and the GRACE-3 Guidelines [14], have been proposed to help differentiate central causes of dizziness. However, these approaches require highly trained clinicians for accurate interpretation and may be difficult to perform in patients experiencing severe dizziness. Moreover, cerebellar function tests (CFTs) have been reported to have low sensitivity for detecting ACI [11]. Therefore, beyond relying solely on physical examination findings, there is a need for objective predictive indicators that can rapidly identify patients at high risk of ACI during the early stage of emergency department evaluation.

Certain medications may influence ACI incidence in IVD patients. Antiplatelet and anticoagulant therapies are intended to prevent cerebrovascular and cardiovascular ischemic events. Current consensus guidelines strongly emphasize the optimal use of these antithrombotic therapies, alongside comprehensive lifestyle modifications—such as smoking cessation, diet optimization, and regular physical activity—as the cornerstone of secondary stroke prevention [15,16]. Based on this established preventive role, one hypothesis is that the incidence of ACI would be lower among patients receiving these medications compared to those who are not. Conversely, because the use of such therapies indicates an existing high risk of ischemic events, an opposite hypothesis is that the incidence of ACI would be higher among these patients. To date, no study has specifically addressed this issue.

This study aimed to assess how frequently ACI occurs in patients presenting with IVD and to evaluate whether the use of antiplatelet or anticoagulant therapy is associated with ACI incidence. In this study, IVD was defined as dizziness without any neurological deficit other than abnormal CFT findings, consistent with the authors’ previous study.

## Materials and methods

### Study design and setting‌‌

We conducted a retrospective cohort study. Ethical approval for this study was obtained from the Institutional Review Board (IRB) of VHS Medical Center (IRB No. 2025-03-013). Due to its observational design, the IRB granted a waiver of informed consent for all individuals included in this study. The data were accessed for research purposes from 30/06/2025 to 12/04/2026, in accordance with IRB approval. In accordance with IRB recommendations, the study was conducted with strict attention to maintaining patient confidentiality throughout the study period. This study adhered to the ethical standards outlined in the Declaration of Helsinki, originally adopted in 1975 and amended in 2000. The Standards for Reporting Diagnostic Accuracy (STARD) guidelines were used as a reference for result analysis [17,18].

The study was performed at a tertiary care center located in an urban area with a capacity of 1,200 inpatient beds. The emergency department manages roughly 30,000 patient visits per year and is continuously staffed by board-certified emergency physicians. These physicians evaluated patients who presented with dizziness as their chief complaint during triage. Neurology consultations and brain imaging were available around the clock.

### Participants

Adult patients (aged ≥18 years) who presented to the ED with a chief complaint of dizziness or vertigo during the four-year study period (January 1, 2019 to December 31, 2022) were assessed for eligibility. For the purpose of this study, IVD was characterized as dizziness occurring in the absence of altered consciousness, visual disturbances such as diplopia, language or speech abnormalities (e.g., aphasia or dysarthria), cranial nerve dysfunction including facial weakness, motor or sensory impairments, or identifiable secondary causes such as active infection, metabolic imbalance, recent physical trauma, allergic reaction, substance intoxication, or comparable etiologies. According to this definition, abnormal CFT results—such as heel-to-shin, finger-to-nose, rapid alternating movement, tandem gait, gait disturbance, and a positive Romberg’s test—were not considered exclusion criteria.

**Fig 1:**
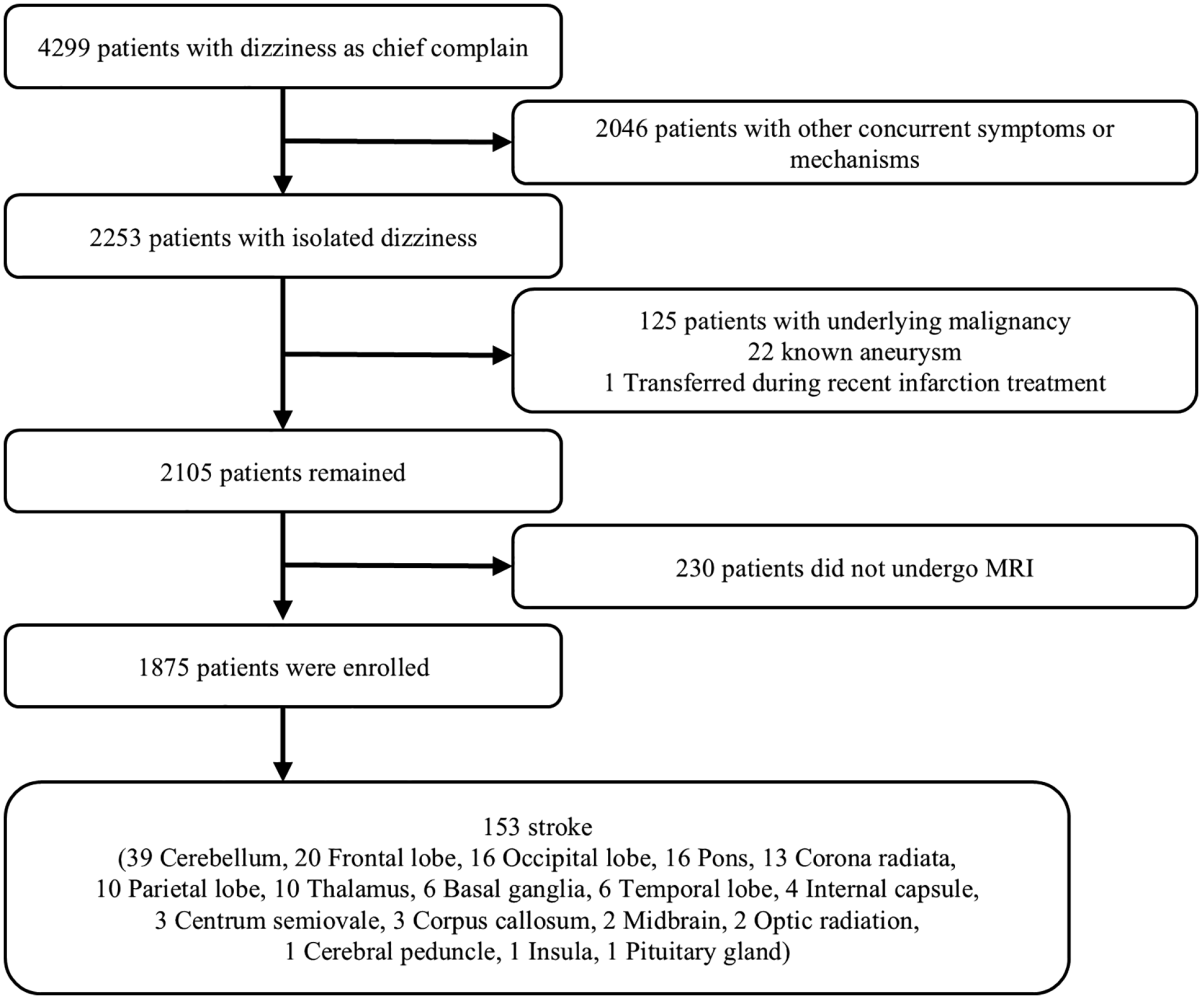
Flow of the study.

Exclusion criteria were as follows: (1) patients with other concurrent symptoms or secondary mechanisms; (2) patients with a history of underlying malignancy or aneurysm, or those who were transferred while undergoing treatment for a recent infarction; and (3) patients who did not undergo MRI. The second exclusion condition was applied to minimize potential sources of bias. A total of 4,299 ED patients were screened, and 2,046 were excluded for not meeting the IVD definition. Additionally, patients with malignancy (n = 125), aneurysm (n = 22), or transfer during recent infarction treatment (n = 1) were excluded. Among the remaining patients, 230 did not undergo MRI and were therefore excluded from the analysis. As a result, 1,875 patients were included in the final analysis (Fig 1).

### Data source and variables

Researchers accessed the study hospital’s electronic medical records (EMRs). The EMRs were reviewed by a trained researcher in accordance with the guidelines recommended by Gilbert et al [19]. A board-certified emergency medicine specialist reviewed the records, and subsequent chart reviews were supervised by the corresponding author, who is also a board-certified emergency medicine specialist. All researchers received regular training in research ethics and data acquisition based on institutional standards.

Data were collected on the following variables: sex, age, emergency medical service (EMS) use, comorbidities—including hypertension (HTN), diabetes mellitus (DM), dyslipidemia, cerebrovascular disease (defined as a documented history of ischemic stroke, intracerebral hemorrhage, or transient ischemic attack), chronic kidney disease, coronary artery disease, atrial fibrillation (Af), chronic obstructive pulmonary disease (COPD), and asthma—vital signs, and dizziness characteristics (spinning/whirling sensation, positional vertigo/dizziness, presence of nystagmus, continuous dizziness, duration, and time to onset). Initial laboratory results included D-dimer, hemoglobin, and glucose levels. Additional data were collected on the Trial of Org 10172 in Acute Stroke Treatment (TOAST) classification, hospitalization status, and bMRI-DWI results. Information on the use of antiplatelet and anticoagulant medications, including medication names, was also obtained. The terminology for dizziness characteristics was defined with reference to the Bárány Society classification and the TiTRATe framework [20,21]. According to the TiTRATe framework, continuous dizziness refers to dizziness that persists for days to weeks, in contrast to episodic dizziness.

### Main outcome

The primary outcome was the presence of ACI, defined as a lesion identified on bMRI-DWI. Only MRI-confirmed ACIs were considered as outcome events in this study. At the study hospital, bMRI was typically performed within 2 hours of ED presentation. DWI was performed using a 3-Tesla MRI scanner (Skyra, Siemens, Germany) with a slice thickness of 5 mm and an interslice gap of 2 mm. All DWI images were independently reviewed and confirmed by a board-certified radiologist.

### Statistical analysis

Continuous variables were summarized using mean ± standard deviation (SD) when normally distributed, and median with interquartile range (IQR) when distributions were non-normal. Categorical variables were reported as counts and percentages. Statistical significance was defined as a two-sided p value of <0.05.

Normally distributed variables were compared using the independent samples t-test or one-way analysis of variance (ANOVA). Non-normally distributed variables were compared using the Mann–Whitney U test or Kruskal–Wallis test. For categorical variables, the chi-square test was used, with Fisher’s exact test applied when appropriate.

To evaluate the diagnostic performance of medication group classification in identifying patients with ACI, a 2 × 2 contingency table was constructed based on the presence or absence of ACI across medication groups. Using this table, we calculated sensitivity, specificity, positive predictive value (PPV), negative predictive value (NPV), accuracy, positive likelihood ratio (PLR), and negative likelihood ratio (NLR). Additionally, discriminatory ability was assessed using receiver operating characteristic (ROC) curve analysis, and the area under the curve (AUC) was calculated to summarize classification performance across all possible thresholds. These diagnostic metrics were used to assess the predictive value of medication groups prior to conducting logistic regression analysis.

Univariable logistic regression analyses were initially conducted to assess the associations between ACI and each independent variable. Variables with p < 0.1 were considered candidates for multivariable logistic regression. We evaluated multicollinearity using the Generalized Variance Inflation Factor (GVIF). For variables with more than one degree of freedom, adjusted GVIF values were calculated as GVIF^(1/(2 × df)). All adjusted GVIF values were below 2, suggesting that multicollinearity was not a concern in the final models.

Regression results are presented as odds ratios (ORs) with 95% confidence intervals (CIs). The primary independent variable was the patient’s use of antiplatelet or anticoagulant medication. Two models were used to categorize this variable. Model 1 included three groups: a no-medication group (no antiplatelet or anticoagulant), an antiplatelet group (aspirin or clopidogrel), and an anticoagulant group (warfarin or non–vitamin K antagonist oral anticoagulants [NOACs], regardless of antiplatelet use). Model 2 provided a more detailed classification: no-medication, aspirin-only, clopidogrel-only, dual antiplatelet therapy (aspirin and clopidogrel), and anticoagulant.

Given that medication use may be confounded by comorbidities such as cerebrovascular disease, coronary artery disease, or atrial fibrillation, we performed multivariable logistic regression analyses in two ways: Model A without adjustment for comorbidities, and Model B with adjustment. In total, four logistic regression models (Models 1A, 1B, 2A, and 2B) were constructed.

Although the effect of individual medications was assessed through univariable logistic regression, individual medications were not included in the multivariable models. To test the robustness of the medication effect and assess potential effect modification, subgroup analyses were conducted for each covariate included in Model B. Both categorical and continuous variables were considered; continuous variables were dichotomized using median values. In each subgroup analysis, the stratifying variable was excluded from the adjustment set to avoid overadjustment, and the effect of medication on ACI was estimated using logistic regression adjusted for the remaining covariates.

Additionally, sensitivity analyses were performed using TOAST classification-based subtypes of ACI as alternative outcome variables. For each subtype, a separate multivariable logistic regression model was fitted using the same covariates as Model B. When analyzing a given subtype, patients with other subtypes were treated as missing.

All analyses were performed using Stata 11.1 (StataCorp LLC, College Station, TX, USA) and R version 4.3.2 (R Foundation for Statistical Computing, Vienna, Austria)

## Results

### Characteristics of enrolled patients

Baseline characteristics of the study population are presented in Table 1. A total of 1,875 patients were included in the study. The mean age was 74.4 ± 9.5 years, and 1,342 (71.6%) were male. Hypertension was the most common comorbidity (64.0%), followed by diabetes mellitus (21.2%), cerebrovascular disease (17.2%), dyslipidemia (14.8%), and coronary artery disease (14.4%). Regarding medication use, 531 patients (28.3%) took aspirin only, 298 (15.9%) took clopidogrel only, 687 (36.6%) took aspirin or clopidogrel, 12 (0.6%) took warfarin, and 89 (4.7%) took non–vitamin K antagonist oral anticoagulants (NOACs).

**Table 1:** Baseline characteristics of enrolled patients and comparison between ACI and non-ACI group.

Variable	All (n = 1875)	Non-ACI (n = 1722)	ACI (n = 153)	P value
Age, year	74.4 ± 9.5	74.0 [71.0;79.0]	76.0 [73.0;83.0]	<0.001
**Age groups, n (%)**				0.005
<65	203 (10.8)	194 (11.3)	9 (5.9)	
65-74	745 (39.7)	695 (40.4)	50 (32.7)	
≥75	927 (49.4)	833 (48.4)	94 (61.4)	
Male, n (%)	1342 (71.6)	1212 (70.4)	130 (85.0)	<0.001
EMS use, n (%)	309 (16.5)	278 (16.1)	31 (20.3)	0.229
**Comorbidity, n (%)**				
Hypertension	1200 (64.0)	1102 (64.0)	98 (64.1)	1.000
Diabetes mellitus	397 (21.2)	356 (20.7)	41 (26.8)	0.094
Dyslipidemia	277 (14.8)	241 (14.0)	36 (23.5)	0.002
Cerebrovascular disease	323 (17.2)	275 (16.0)	48 (31.4)	<0.001
Chronic kidney disease	99 (5.3)	89 (5.2)	10 (6.5)	0.592
Coronary artery disease	270 (14.4)	247 (14.3)	23 (15.0)	0.910
Atrial fibrillation	70 (3.7)	58 (3.4)	12 (7.8)	0.010
COPD/ Asthma	72 (3.8)	69 (4.0)	3 (2.0)	0.297
**Physiology**				
SBP, mmHg	150.1 ± 26.4	149.0 [131.0;168.0]	152.0 [131.0;171.0]	0.495
DBP, mmHg	86.8 ± 16.4	86.0 [77.0;96.0]	86.0 [76.0;96.0]	0.896
PR, bpm	77.6 ± 14.8	76.0 [67.0;86.0]	79.0 [70.0;86.0]	0.096
RR, bpm	18.9 ± 1.6	20.0 [18.0;20.0]	20.0 [18.0;20.0]	0.067
BT, ˚C	36.5 ± 0.2	36.5 [36.4;36.6]	36.5 [36.4;36.6]	0.504
NEWS2	0.6 ± 0.9	0.0 [0.0; 1.0]	0.0 [0.0; 1.0]	0.024
SpO₂, %	97.3 ± 1.5	98.0 [96.0;98.0]	98.0 [96.0;98.0]	0.623
Admission, n (%)	164 (8.7)	80 (4.6)	84 (54.9)	<0.001
**Dizziness feature, n (%)**				
Spinning/Whirling	589 (31.4)	558 (32.4)	31 (20.3)	0.003
Positional vertigo/dizziness	907 (48.4)	850 (49.4)	57 (37.3)	0.005
Any nystagmus	73 (3.9)	70 (4.1)	3 (2.0)	0.284
Continuous (vs episodic) dizziness	1254 (66.9)	1133 (65.8)	121 (79.1)	0.001
**Duration of symptom, n (%)**				0.002
<10 min	573 (30.6)	542 (31.5)	31 (20.3)	
10-59 min	33 (1.8)	33 (1.9)	0 (0.0)	
≥60 min	1269 (67.7)	1147 (66.6)	122 (79.7)	
Time to onset, hr	190.4 ± 707.4	24.0 [5.0;144.0]	24.0 [7.0;72.0]	0.891
D-dimer, mg/L (n = 1846)	0.8 ± 1.5	0.4 [0.3; 0.8]	0.6 [0.4; 1.1]	<0.001
Hemoglobin	13.3 ± 1.6	13.4 [12.4;14.4]	13.8 [12.4;14.8]	0.073
Glucose	128.8 ± 27.8	123.0 [108.0;147.0]	127.0 [108.0;150.0]	0.413
**TOAST classification (n = 153), n (%)**				
Large artery atherosclerosis	25 (1.3)	0 (0.0)	25 (16.3)	
Cardioembolism	8 (0.4)	0 (0.0)	8 (5.2)	
Small-vessel occlusion	66 (3.5)	0 (0.0)	66 (43.1)	
Stroke of other determined etiology	1 (0.1)	0 (0.0)	1 (0.7)	
Stroke of undetermined etiology	53 (2.8)	0 (0.0)	53 (34.6)	
**Medication, n (%)**				
Aspirin	531 (28.3)	482 (28.0)	49 (32.0)	0.333
Clopidogrel	298 (15.9)	266 (15.4)	32 (20.9)	0.097
Aspirin or Clopidogrel	687 (36.6)	627 (36.4)	60 (39.2)	0.547
Warfarin	12 (0.6)	12 (0.7)	0 (0.0)	0.615
Non-vitamin K oral anticoagulant (NOAC)	89 (4.7)	69 (4.0)	20 (13.1)	<0.001
Warfarin or NOAC	101 (5.4)	81 (4.7)	20 (13.1)	<0.001
**Three groups, n (%)**				<0.001
No-medication	1104 (58.9)	1030 (59.8)	74 (48.4)	
Antiplatelet	670 (35.7)	611 (35.5)	59 (38.6)	
Anticoagulant	101 (5.4)	81 (4.7)	20 (13.1)	
**Five groups, n (%)**				<0.001
No-medication	1104 (58.9)	1030 (59.8)	74 (48.4)	
Aspirin	377 (20.1)	349 (20.3)	28 (18.3)	
Clopidogrel	152 (8.1)	142 (8.2)	10 (6.5)	
Aspirin and Clopidogrel	141 (7.5)	120 (7.0)	21 (13.7)	
Anticoagulant	101 (5.4)	81 (4.7)	20 (13.1)	

Abbreviation. EMS, emergency medical service; COPD, chronic obstructive pulmonary disease; SBP, systolic blood pressure; DBP, diastolic blood pressure; PR, pulse rate; RR, respiratory rate; BT, body temperature; NEWS2, national early warning score 2; SpO2, peripheral capillary oxygen saturation; ICU, intensive care unit; min, minute; hr, hour; mg/L, milligrams per liter; TOAST, trial of ORG 10172 in acute stroke treatment. Continuous variables are presented as mean ± SD or median [IQR].

Table 1 also compares the baseline characteristics between the ACI and non-ACI groups. Among the 1,875 patients, 153 (8.2%) were diagnosed with ACI. The median age was higher in the ACI group than in the non-ACI group. The proportion of male patients was also higher in the ACI group (85.0% vs. 70.4%). ACI patients had higher rates of cerebrovascular disease (31.4% vs. 16.0%), dyslipidemia (23.5% vs. 14.0%), and atrial fibrillation (7.8% vs. 3.4%). However, there was no significant difference in the prevalence of coronary artery disease (15.0% vs. 14.3%). In terms of patient disposition, general ward admission was much more common in the ACI group than in the non-ACI group (54.9% vs. 4.6%). Regarding dizziness characteristics, the non-ACI group had higher proportions of spinning/whirling sensations (32.4% vs. 20.3%) and positional vertigo/dizziness (49.4% vs. 37.3%), whereas the ACI group had a higher rate of continuous dizziness (79.1% vs. 65.8%). The median D-dimer level was higher in ACI patients (0.6 mg/L [0.4–1.1] vs. 0.4 mg/L [0.3–0.8]). Among the 153 patients with ACI, the TOAST classification revealed that the most common etiology was small-vessel occlusion (43.1%), followed by stroke of undetermined etiology (34.6%), large-artery atherosclerosis (16.3%), cardioembolism (5.2%), and other determined etiologies (0.7%).

The ACI group had higher use of antiplatelets (38.6% vs. 35.5%) and anticoagulants (13.1% vs. 4.7%). Patients who took aspirin only (20.3% vs. 18.3%) and clopidogrel only (8.2% vs. 6.5%) were more frequent in the non-ACI group, while those on dual antiplatelet therapy were more common in the ACI group (13.7% vs. 7.0%). The anatomical locations of the 153 infarctions are presented in Fig 1. The cerebellum was the most commonly affected site, followed by the frontal lobe, occipital lobe, pons, and corona radiata. S1 and S2 Tables present the comparison of characteristics among medication subgroups.

Based on a 2 × 2 contingency table comparing medication use with ACI status, anticoagulant use showed an AUC of 0.54 (95% CI: 0.51–0.57), with low sensitivity (0.13; 95% CI: 0.08–0.19) but high specificity (0.95; 95% CI: 0.94–0.96) and a positive likelihood ratio of 2.78 (95% CI: 1.75–4.40). dual antiplatelet therapy demonstrated an AUC of 0.53 (95% CI: 0.51–0.56), sensitivity of 0.14 (95% CI: 0.09–0.20), specificity of 0.93 (95% CI: 0.92–0.94), and a PLR of 1.97 (95% CI: 1.28–3.04). Although the discriminative ability of medication use alone was limited, both anticoagulant use and dual antiplatelet therapy showed high specificity with modest positive likelihood ratios (Table 2).

**Table 2:** Area under the receiver operating characteristic curve, sensitivity, specificity, diagnostic accuracy, positive predictive value, negative predictive value, positive likelihood ratio, negative likelihood ratio of each subgroup of the study. Values were presented with 95% confidence interval.

Group	AUC	Sensitivity	Specificity	Diagnostic accuracy	PPV	NPV	PLR	NLR
Anticoagulant	0.54 (0.51-0.57)	0.13 (0.08-0.19)	0.95 (0.94-0.96)	0.89 (0.87-0.90)	0.20 (0.13-0.29)	0.93 (0.91-0.94)	2.78 (1.75-4.40)	0.91 (0.86-0.97)
Antiplatelet	0.52 (0.48-0.56)	0.39 (0.31-0.47)	0.65 (0.62-0.67)	0.62 (0.60-0.65)	0.09 (0.07-0.11)	0.92 (0.91-0.94)	1.09 (0.88-1.34)	0.95 (0.84-1.08)
Aspirin and Clopidogrel	0.53 (0.51-0.56)	0.14 (0.09-0.20)	0.93 (0.92-0.94)	0.87 (0.85-0.88)	0.15 (0.09-0.22)	0.92 (0.91-0.94)	1.97 (1.28-3.04)	0.93 (0.87-0.99)
Aspirin-only	0.49 (0.46-0.52)	0.18 (0.13-0.25)	0.80 (0.78-0.82)	0.75 (0.73-0.77)	0.07 (0.05-0.11)	0.92 (0.90-0.93)	0.90 (0.64-1.28)	1.02 (0.95-1.11)
Clopidogrel-only	0.49 (0.47-0.51)	0.07 (0.03-0.12)	0.92 (0.90-0.93)	0.85 (0.83-0.86)	0.07 (0.03-0.12)	0.92 (0.90-0.93)	0.79 (0.43-1.47)	1.02 (0.97-1.06)

Abbreviation. AUC, Area under the receiver operating characteristic curve; PPV, Positive predictive value; NPV, Negative predictive value; PLR, Positive likelihood ratio; NLR, Negative likelihood ratio.

**Fig 2:**
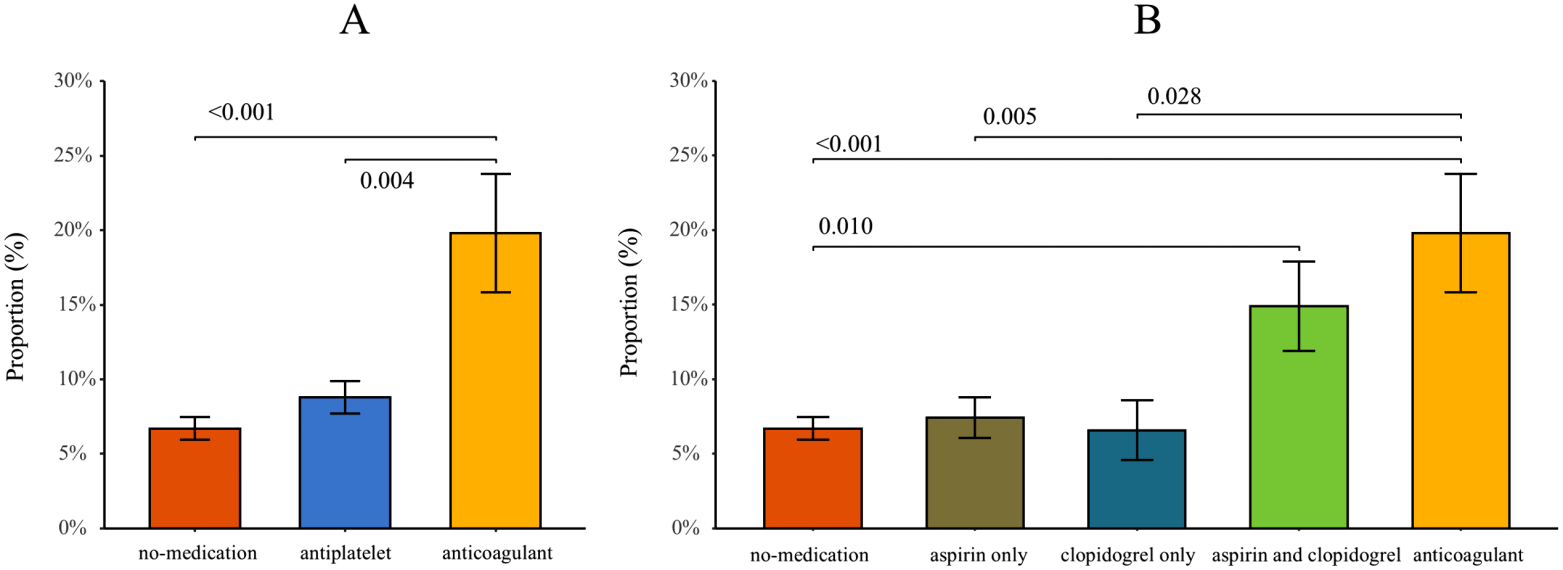
Comparison of ACI incidence among medication groups. **(A)** Comparison of ACI incidence among no-medication, antiplatelet, and anticoagulant groups. **(B)** Comparison of ACI incidence among no-medication, aspirin-only, clopidogrel-only, aspirin and clopidogrel, and anticoagulant groups. Bar plots represent the incidence of ACI in each group, with error bars indicating standard errors. Post hoc comparisons were conducted using the Chi-square test or Fisher’s exact test, and p-values were adjusted using the Bonferroni correction. Only statistically significant p-values are‌‌ displayed above the relevant comparisons. Abbreviations. ACI, acute cerebral infarction.

Fig 2 shows the comparison of ACI incidence among medication subgroups. Panel A compares the incidence of ACI among the no-medication, antiplatelet, and anticoagulant groups. Panel B compares ACI incidence among the no-medication, aspirin-only, clopidogrel-only, dual antiplatelet therapy, and anticoagulant groups. The incidence of ACI was 6.7% (74/1,104) in the no-medication group, 8.8% (59/670) in the antiplatelet group, and 19.8% (20/101) in the anticoagulant group. The incidence in the anticoagulant group was significantly higher than that in the no-medication and antiplatelet groups (Fig 2A). ACI incidence was 7.4% (28/377) in the aspirin-only group, 6.6% (10/152) in the clopidogrel-only group, and 14.9% (21/141) in the dual antiplatelet therapy group. The anticoagulant group showed significantly higher ACI incidence than the no-medication, aspirin-only, and clopidogrel-only groups (Fig 2B). Additionally, the incidence in the dual antiplatelet therapy group was significantly higher than that in the no-medication group (Fig 2B).

### Logistic regression analysis

Statistical significance of individual variables was initially assessed using univariable logistic regression analysis. Variables that showed statistical significance or a trend toward significance included age, male sex, diabetes mellitus, dyslipidemia, cerebrovascular disease, atrial fibrillation, NEWS2 score, spinning/whirling sensation, positional vertigo/dizziness, continuous dizziness, symptom duration, D-dimer level, anticoagulant group, and dual antiplatelet therapy group. These variables were considered for inclusion in the multivariable models. Among them, symptom duration was excluded due to multicollinearity. The remaining variables were included in the multivariable logistic regression analyses.

In Model 1A, the anticoagulant group remained a significant predictor (adjusted odds ratio [AOR], 2.52; 95% confidence interval [CI], 1.44–4.41; p = 0.001), while the antiplatelet group did not. In Model 2A, both the anticoagulant group and the dual antiplatelet therapy group were significant (AOR, 2.53; 95% CI, 1.45–4.44; p = 0.001, and AOR, 1.74; 95% CI, 1.02–2.97; p = 0.044, respectively), while the aspirin-only and clopidogrel-only groups were not.

In Model 1B, the anticoagulant group remained significant (AOR, 2.47; 95% CI, 1.20–5.08; p = 0.014), whereas the antiplatelet group did not. Similarly, in Model 2B, the anticoagulant group remained significant (AOR, 2.46; 95% CI, 1.20–5.08; p = 0.014), while the aspirin-only, clopidogrel-only, and dual antiplatelet therapy groups were not.

Among co-variants, age, male sex, and continuous dizziness were significant predictors, whereas NEWS2 score, other dizziness characteristics (excluding continuous dizziness), and D-dimer level were not. Among comorbidities, dyslipidemia and cerebrovascular disease remained significant, while DM and atrial fibrillation did not (Table 3).

**Table 3:** Multivariable logistic regression models assessing the association between antithrombotic medication and acute cerebral infarction.

Variable	Univariate	Multivariate model 1 A	Multivariate model 1 B	Multivariate model 2 A	Multivariate model 2 B
Age, year	1.04 (1.02-1.06, < 0.001)	1.03 (1.01-1.05, 0.009)	1.03 (1.01-1.05, 0.008)	1.03 (1.01-1.05, 0.009)	1.03 (1.01-1.05, 0.007)
**Age groups**					
<65	Reference				
65-74	1.55 (0.75-3.21, 0.237)				
≥75	2.43 (1.21-4.91, 0.013)				
Male	2.38 (1.51-3.75, < 0.001)	2.08 (1.31-3.33, 0.002)	2.08 (1.30-3.34, 0.002)	2.01 (1.26-3.22, 0.004)	2.01 (1.25-3.24, 0.004)
EMS use	1.32 (0.87-2.00, 0.190)				
**Comorbidity**					
Hypertension	1.00 (0.71-1.41, 0.989)				
Diabetes mellitus	1.40 (0.96-2.05, 0.077)		1.17 (0.79-1.74, 0.431)		1.18 (0.79-1.75, 0.420)
Dyslipidemia	1.89 (1.27-2.81, 0.002)		1.92 (1.26-2.93, 0.002)		1.92 (1.26-2.93, 0.002)
Cerebrovascular disease	2.41 (1.67-3.47, < 0.001)		2.00 (1.36-2.94, < 0.001)		1.97 (1.34-2.90, 0.001)
Chronic kidney disease	1.28 (0.65-2.52, 0.470)				
Coronary artery disease	1.06 (0.66-1.68, 0.816)				
Atrial fibrillation	2.44 (1.28-4.65, 0.007)		0.82 (0.34-1.98, 0.661)		0.84 (0.35-2.02, 0.689)
COPD/ Asthma	0.48 (0.15-1.54, 0.217)				
**Physiology**					
SBP, mmHg	1.00 (1.00-1.01, 0.393)				
DBP, mmHg	1.00 (0.99-1.01, 0.802)				
PR, bpm	1.01 (1.00-1.02, 0.130)				
RR, bpm	1.09 (0.98-1.21, 0.118)				
BT, ˚C	1.07 (0.54-2.13, 0.836)				
NEWS2	1.22 (1.03-1.44, 0.019)	1.18 (0.99-1.40, 0.063)	1.18 (0.99-1.40, 0.058)	1.18 (0.99-1.40, 0.066)	1.18 (0.99-1.40, 0.062)
SpO₂, %	0.96 (0.85-1.07, 0.430)				
Admission	24.99 (16.92-36.89, < 0.001)				
**Dizziness feature**					
Spinning/Whirling	0.53 (0.35-0.80, 0.002)	0.67 (0.40-1.12, 0.127)	0.68 (0.41-1.14, 0.142)	0.69 (0.41-1.14, 0.148)	0.70 (0.42-1.16, 0.167)
Positional vertigo/dizziness	0.61 (0.43-0.86, 0.004)	1.00 (0.63-1.59, 0.989)	1.02 (0.63-1.63, 0.948)	0.99 (0.62-1.58, 0.966)	1.00 (0.62-1.61, 0.992)
Any nystagmus	0.472 (0.15-1.52, 0.208)				
Continuous (vs episodic) dizziness	1.97 (1.31-2.94, 0.001)	1.65 (1.05-2.59, 0.031)	1.67 (1.06-2.64, 0.029)	1.61 (1.02-2.53, 0.040)	1.64 (1.03-2.60, 0.035)
**Duration of symptom**					
<10 min					
10-59 min	Empty				
≥60 min	1.86 (1.24-2.79, 0.003)				
Time to onset, hr	1.00 (1.00-1.00, 0.142)				
D-dimer, mg/L (n = 1846)	1.07 (0.99-1.16, 0.095)	1.05 (0.96-1.14, 0.285)	1.06 (0.97-1.16, 0.178)	1.05 (0.96-1.15, 0.285)	1.06 (0.97-1.16, 0.182)
Hemoglobin	1.09 (0.98-1.21, 0.128)				
Glucose	1.00 (1.00-1.01, 0.435)				
**Medication**					
Aspirin	1.21 (0.85-1.73, 0.289)				
Clopidogrel	1.45 (0.96-2.18, 0.078)				
Aspirin or Clopidogrel	1.13 (0.80-1.58, 0.490)				
Warfarin	omit				
Non-vitamin K oral anticoagulant (NOAC)	3.60 (2.12-6.11, < 0.001)				
Warfarin or NOAC	3.04 (1.81-5.13, < 0.001)				
**Three groups**					
No-medication	Reference	Reference	Reference		
Antiplatelet	1.34 (0.94-1.92, 0.104)	1.06 (0.74-1.53, 0.745)	0.85 (0.58-1.25, 0.409)		
Anticoagulant	3.44 (2.00-5.92, < 0.001)	2.52 (1.44-4.41, 0.001)	2.47 (1.20-5.08, 0.014)		
**Five groups**					
No-medication	Reference			Reference	Reference
Aspirin	1.12 (0.71-1.75, 0.632)			0.92 (0.58-1.46, 0.715)	0.75 (0.46-1.20, 0.233)
Clopidogrel	0.98 (0.49-1.94, 0.954)			0.80 (0.40-1.59, 0.519)	0.64 (0.31-1.29, 0.209)
Aspirin and Clopidogrel	2.44 (1.45-4.10, 0.001)			1.74 (1.02-2.97, 0.044)	1.34 (0.77-2.34, 0.297)
Anticoagulant	3.44 (2.00-5.92, < 0.001)			2.53 (1.45-4.44, 0.001)	2.46 (1.20-5.08, 0.014)

Model 1 categorized patients into three groups: no medication, antiplatelet therapy, and anticoagulant therapy.

Model 2 included five groups: no medication, aspirin-only, clopidogrel-only, dual antiplatelet therapy, and anticoagulant therapy. For each, Model A was adjusted for age, sex, National Early Warning Score 2 (NEWS2), presence of whirling sensation, positional vertigo/dizziness, continuous dizziness, and D-dimer level (without adjustment for comorbidities), while Model B was additionally adjusted for diabetes mellitus, dyslipidemia, cerebrovascular disease, and atrial fibrillation (with adjustment for comorbidities).

Odds ratios (ORs) and 95% confidence intervals (CIs) are reported.

Abbreviation. EMS, emergency medical service; COPD, chronic obstructive pulmonary disease; SBP, systolic blood pressure; DBP, diastolic blood pressure; PR, pulse rate; RR, respiratory rate; BT, body temperature; NEWS2, national early warning score 2; SpO2, peripheral capillary oxygen saturation; ICU, intensive care unit; min, minute; hr, hour; mg/L, milligrams per liter.

Subgroup analyses revealed generally consistent medication effects with the primary findings, although some variability was observed, likely due to smaller sample sizes in certain subgroups (S1 and S2 Figs). In the TOAST-based analysis, the medication effect was consistent with the main findings only in the undetermined (UD) subtype. No significant differences were observed in the other TOAST subtypes (S3 and S4 Figs).

## Discussion

The incidence of ACI among patients with IVD has been evaluated in numerous studies over time. An earlier study from 2006 reported a relatively low incidence of 0.7% [7]. However, more recent studies have reported higher incidences, ranging from 3% to 17% [5,6,8–11]. Taken together, these findings suggest that ACI is not a rare occurrence and should be considered a clinically important risk among IVD patients. The presence of traditional cerebrovascular risk factors—such as DM, dyslipidemia, and prior cerebrovascular or cardiovascular events—should raise the clinical suspicion of ACI in patients presenting with IVD.

In the present study, the overall incidence of ACI was 8.2% among ED patients with IVD. Although no standardized guidelines exist for when to perform MRI in IVD patients, this incidence represents a clinically meaningful risk, underscoring the importance of risk stratification to guide selective imaging. Furthermore, this risk appeared to be more pronounced in specific medication groups. The incidence was significantly higher in the anticoagulant group (19.8%) compared to the no-medication group (6.7%) and the antiplatelet group (8.8%). Additionally, the dual antiplatelet therapy group exhibited a significantly higher incidence of ACI (14.9%) compared to the no-medication group. There were no significant differences in incidence among the no-medication, aspirin-only, and clopidogrel-only groups.

In addition, diagnostic performance analysis showed that anticoagulant use and dual antiplatelet therapy demonstrated limited discriminative ability for identifying ACI (AUC 0.54 and 0.53, respectively). However, both variables showed relatively high specificity and modest positive likelihood ratios, suggesting that medication history may still serve as a useful clinical indicator when evaluating patients with IVD.

In the multivariable analysis of this study, increasing age, male sex, dyslipidemia, a history of cerebrovascular disease, and continuous dizziness were significantly associated with the occurrence of acute cerebral infarction (ACI). These variables represent well-established clinical indicators of stroke reported in previous studies [22,23], and our findings reaffirm their clinical importance. In Model 2A, both anticoagulant use and dual antiplatelet therapy were significantly associated with an increased risk of ACI. Furthermore, anticoagulant use remained a statistically significant independent predictor of ACI even in Model 2B, after adjusting for comorbidities. The persistence of this association suggests that anticoagulant use may serve as a surrogate marker of a higher underlying vascular risk burden. This elevated baseline vascular risk may not be fully captured by simply recording the presence of comorbid conditions. Therefore, integrating the history of antithrombotic use identified in this study with these conventional risk factors may provide a practical basis for developing a risk stratification strategy to more accurately and rapidly identify high-risk patients in the early stages of emergency department presentation.

Antiplatelet and anticoagulant therapies are widely used to prevent cerebrovascular and cardiovascular thrombotic events [24–26]. This raises two competing hypotheses: (1) that medication use lowers ACI incidence due to its preventive effects; or (2) that medication use identifies patients already at high risk for thrombotic events, resulting in higher observed ACI incidence. This study aimed to evaluate these competing hypotheses.

We found that the ACI incidence was highest among IVD patients who were taking anticoagulants (19.8%), followed by those on dual antiplatelet therapy (14.9%). These findings suggest that both groups should be considered high-risk populations. Their medication status may serve as a useful proxy for clinicians when assessing the need for neuroimaging in IVD patients in the ED setting.

Anticoagulation is a commonly used preventive strategy for ischemic stroke or transient ischemic attack (TIA) in patients with non-valvular atrial fibrillation, left ventricular thrombus, acute myocardial infarction, rheumatic mitral valve disease, and prosthetic heart valves. These conditions are well-known risk factors for embolic stroke, which may partly explain the high ACI incidence observed in the anticoagulant group. Furthermore, consistent with our multivariable findings, the requirement for anticoagulation likely reflects a more advanced stage of these underlying conditions, conferring a high embolic risk that may not be fully captured by the recorded diagnosis alone.

Of note, dual antiplatelet therapy is also frequently prescribed as a preventive strategy. However, several studies, including the MATCH trial [27], CHARISMA trial [28], and SPS3 trial [29], have raised concerns about its long-term use. Current guidelines recommend dual antiplatelet therapy primarily for specific indications such as acute coronary syndrome (ACS), percutaneous coronary intervention (PCI) with drug-eluting stent (DES), revascularization for stable ischemic heart disease or peripheral artery disease (PAD), and symptomatic carotid artery stenosis [30–32].

Given the strong association between cerebrovascular and coronary artery disease—both of which are manifestations of systemic atherosclerosis—the relatively high incidence of ACI in the dual antiplatelet therapy group may reflect the net effect of underlying comorbidities outweighing the potential benefits of the therapy. In our cohort, 45.4% of patients in the aspirin and clopidogrel group had coronary artery disease and 35.5% had cerebrovascular disease (S2 Table). However, the study data could not determine whether the prescription of dual antiplatelet therapy in these patients was due to prior cardiac or cerebrovascular conditions.

The incidence of ACI among the no-medication, aspirin-only, and clopidogrel-only groups did not differ significantly, and adjusted odds ratios (AORs) were also not significantly different across these groups. A potential explanation is the balance between risk and benefit: the harmful impact of underlying cardiovascular ischemic disease [33,34] may be offset by the protective effect of antiplatelet therapy. Consequently, the ACI incidence in the aspirin-only and clopidogrel-only groups was comparable to that in the no-medication group.

Importantly, the higher incidence of ACI in the anticoagulant and dual antiplatelet therapy groups does not imply the need for more aggressive dosing, prolonged therapy, or increased treatment intensity. Clinicians must carefully consider the bleeding risks associated with these therapies, including life-threatening complications such as gastrointestinal bleeding, intracranial hemorrhage, and other major bleeding events [35–38]. Current stroke prevention guidelines emphasize that appropriate antithrombotic therapy, together with lifestyle modification and aggressive management of vascular risk factors, remains the cornerstone of secondary stroke prevention [15,16]. Therefore, adherence to current stroke prevention guidelines is essential [25]. In particular, as recently highlighted in a prospective study, optimizing secondary stroke prevention strategies is crucial not only for preventing major strokes but also for alleviating recurrent vertigo and dizziness in patients with vertebrobasilar TIA [39].

Furthermore, the clinical significance of ACI in IVD patients remains debatable, especially in the context of healthcare resource allocation. Savitz et al. previously reported that misdiagnosed cerebellar infarctions carried a 40% mortality rate and that half of the survivors experienced disabling deficits [40]. However, recent studies have suggested that most ACIs in IVD patients are small in size and not accompanied by significant neurological deficits [5,11]. Consistent with prior findings, most infarctions in our cohort were minor in size. These cases may be classified as “minor strokes” [41,42]. Outpatient-based management strategies and specialist clinics have recently been explored as alternatives to short-stay units or inpatient stroke unit care for patients with transient ischemic attack and minor stroke (TIAMS) [43,44].

This study has several notable strengths. The sample size was relatively large compared to prior studies, and the cohort was well refined by excluding confounding medical conditions such as desaturation, anemia, or hypoglycemia that could independently result in isolated dizziness.

However, several limitations should be acknowledged. First, the no-medication group does not represent a healthy population; some patients in this group had comorbidities such as diabetes mellitus, dyslipidemia, or cerebrovascular disease. The reasons for not taking appropriate medications were not explored and may include physician decision or patient noncompliance. Further subgroup analyses, such as among patients with a history of cerebrovascular disease, are warranted. Likewise, evaluating patients without such a history may help clarify whether antiplatelet use is associated with new-onset ACI. Second, this study did not assess medication duration or adherence, both of which may influence the preventive effect of the medications. Third, as a single-center study, these findings may have limited generalizability to other patient populations. The enrolled cohort had specific baseline characteristics, including advanced age, male predominance, and multiple comorbidities. A large, multi-center study is needed to validate these findings. Fourth, the exclusion of patients who did not undergo brain MRI may have introduced selection bias. However, because most patients at the study center were eligible for no-cost or subsidized imaging, the proportion of excluded patients is expected to be lower than in other institutions. Fifth, DWI was performed with a slice thickness of 5 mm and a 2 mm interslice gap, which may reduce sensitivity for detecting small acute infarctions compared with high-resolution DWI protocols with thinner slices and no interslice gap. Sixth, MRI was typically performed within 2 hours of ED presentation. Because DWI obtained early after symptom onset may yield false-negative results, particularly in posterior circulation strokes, some cases of acute cerebral infarction may not have been detected. In addition, because only MRI-confirmed ACIs were included in this study, patients with MRI-negative strokes may have been classified into the non-ACI group. Consequently, the true incidence of acute cerebral infarction in this cohort may have been underestimated. Seventh, unmeasured confounders, such as presyncope, syncope [45], and smoking status [10], may have affected the results. Eighth, because the data were collected by emergency medicine physicians affiliated with the study hospital, the chart review was not fully blinded.

In conclusion, nearly 20% of patients in the anticoagulant group and 15% of those in the dual-antiplatelet group experienced ACI among ED patients with IVD. Anticoagulant use remained a significant predictor of ACI. Although dual antiplatelet use showed significance in unadjusted analyses, its association diminished after adjusting for comorbidities. These two medication groups may serve as clinical proxies for elevated ACI risk in ED patients presenting with IVD.

## Supporting information

S1 Fig Subgroup analysis of the association between medication group (no-medication, antiplatelet, and anticoagulant) and ACI: forest plot of adjusted odds ratios. Covariates included in multivariable model B were used for adjustment, except for the stratifying variable in each subgroup analysis.
(TIF)

S2 Fig Subgroup analysis of the association between detailed medication group (no-medication, aspirin-only, clopidogrel-only, aspirin and clopidogrel, and anticoagulant) and ACI: forest plot of adjusted odds ratios. Covariates included in multivariable model B were used for adjustment, except for the stratifying variable in each subgroup analysis.
(TIF)

S3 Fig Effect of medication group (no-medication, antiplatelet, and anticoagulant) on TOAST subtypes. Forest plot showing the adjusted odds ratios for the association between medication group and each TOAST-defined subtype of ACI. Separate logistic regression models were fitted for each subtype, adjusting for covariates in model B. Patients with other subtypes were treated as missing.
(TIF)

S4 Fig Effect of detailed medication group (no-medication, aspirin-only, clopidogrel-only, aspirin and clopidogrel, and anticoagulant) on TOAST subtypes. Forest plot showing the adjusted odds ratios for the association between medication group and each TOAST-defined subtype of ACI. Separate logistic regression models were fitted for each subtype, adjusting for covariates in model B. Patients with other subtypes were treated as missing.
(TIF)

S1 Table Comparison among no-medication, antiplatelet, anticoagulant group. (DOCX)

S2 Table Comparison among no-medication, aspirin-only group, clopidogrel-only group, aspirin and Clopidogrel group, and anti-coagulant group. (DOCX)

S1 File STROBE_Checklist_IVD_ACI‌‌‌‌. (DOCX)

## References

[1] Schappert SM, Burt CW. Ambulatory care visits to physician offices, hospital outpatient departments, and emergency departments: United States, 2001-02. Vital Health Stat. 2006;13(159):1–66. 16471269

[2] Newman-Toker DE, Cannon LM, Stofferahn ME, Rothman RE, Hsieh Y-H, Zee DS. Imprecision in patient reports of dizziness symptom quality: a cross-sectional study conducted in an acute care setting. Mayo Clin Proc. 2007;82(11):1329–40. doi: 10.4065/82.11.1329 17976352

[3] Newman-Toker DE, Hsieh Y-H, Camargo CA Jr, Pelletier AJ, Butchy GT, Edlow JA. Spectrum of dizziness visits to US emergency departments: cross-sectional analysis from a nationally representative sample. Mayo Clin Proc. 2008;83(7):765–75. doi: 10.4065/83.7.765 18613993 PMC3536475

[4] Lee H. Isolated vascular vertigo. J Stroke. 2014;16(3):124–30. doi: 10.5853/jos.2014.16.3.124 25328871 PMC4200599

[5] Doijiri R, Uno H, Miyashita K, Ihara M, Nagatsuka K. How commonly is stroke found in patients with isolated vertigo or dizziness attack?. J Stroke Cerebrovasc Dis. 2016;25(10):2549–52. doi: 10.1016/j.jstrokecerebrovasdis.2016.06.038 27495834

[6] Happonen T, Nyman M, Ylikotila P, Mattila K, Hirvonen J. Imaging Outcomes of Emergency MR Imaging in Dizziness and Vertigo: A Retrospective Cohort Study. AJNR Am J Neuroradiol. 2024;45(6):819–25. doi: 10.3174/ajnr.A8202 38604735 PMC11288592

[7] Kerber KA, Brown DL, Lisabeth LD, Smith MA, Morgenstern LB. Stroke among patients with dizziness, vertigo, and imbalance in the emergency department: a population-based study. Stroke. 2006;37(10):2484–7. doi: 10.1161/01.STR.0000240329.48263.0d 16946161 PMC1779945

[8] Perloff MD, Patel NS, Kase CS, Oza AU, Voetsch B, Romero JR. Cerebellar stroke presenting with isolated dizziness: Brain MRI in 136 patients. Am J Emerg Med. 2017;35(11):1724–9. doi: 10.1016/j.ajem.2017.06.034 28687453

[9] Sert ET, Cayir S, Mutlu H, Kokulu K. Effectiveness of Clinical Risk Factors in the Detection of Central Pathology in Patients With Isolated Vertigo. J Emerg Med. 2021;60(6):709–15. doi: 10.1016/j.jemermed.2020.12.032 33546921

[10] Zuo L, Zhan Y, Liu F, Chen C, Xu L, Calic Z, et al. Clinical and laboratory factors related to acute isolated vertigo or dizziness and cerebral infarction. Brain Behav. 2018;8(9):e01092. doi: 10.1002/brb3.1092 30099862 PMC6160653

[11] Jo S, Jeong T, Lee JB, Jin Y, Yoon J, Park B. Incidence of acute cerebral infarction or space occupying lesion among patients with isolated dizziness and the role of D-dimer. PLoS One. 2019;14(3):e0214661. doi: 10.1371/journal.pone.0214661 30921431 PMC6438525

[12] Kattah JC, Talkad AV, Wang DZ, Hsieh Y-H, Newman-Toker DE. HINTS to diagnose stroke in the acute vestibular syndrome: three-step bedside oculomotor examination more sensitive than early MRI diffusion-weighted imaging. Stroke. 2009;40(11):3504–10. doi: 10.1161/STROKEAHA.109.551234 19762709 PMC4593511

[13] Vanni S, Pecci R, Casati C, Moroni F, Risso M, Ottaviani M, et al. STANDING, a four-step bedside algorithm for differential diagnosis of acute vertigo in the Emergency Department. Acta Otorhinolaryngol Ital. 2014;34(6):419–26. 25762835 PMC4346998

[14] Edlow JA, Carpenter C, Akhter M, Khoujah D, Marcolini E, Meurer WJ, et al. Guidelines for reasonable and appropriate care in the emergency department 3 (GRACE-3): Acute dizziness and vertigo in the emergency department. Acad Emerg Med. 2023;30(5):442–86. doi: 10.1111/acem.14728 37166022

[15] Kleindorfer DO, Towfighi A, Chaturvedi S, Cockroft KM, Gutierrez J, Lombardi-Hill D, et al. 2021 Guideline for the Prevention of Stroke in Patients With Stroke and Transient Ischemic Attack: A Guideline From the American Heart Association/American Stroke Association. Stroke. 2021;52(7):e364–467. doi: 10.1161/STR.0000000000000375 34024117

[16] Furie KL, Kelly PJ. Secondary Prevention after Ischemic Stroke. N Engl J Med. 2026;394(8):784–92. doi: 10.1056/NEJMcp2415601 41707139

[17] Bossuyt PM, Reitsma JB, Bruns DE, Gatsonis CA, Glasziou PP, Irwig LM, et al. Towards complete and accurate reporting of studies of diagnostic accuracy: the STARD initiative. BMJ. 2003;326(7379):41–4. doi: 10.1136/bmj.326.7379.41 12511463 PMC1124931

[18] Simel DL, Rennie D, Bossuyt PMM. The STARD statement for reporting diagnostic accuracy studies: application to the history and physical examination. J Gen Intern Med. 2008;23(6):768–74. doi: 10.1007/s11606-008-0583-3 18347878 PMC2517891

[19] Gilbert EH, Lowenstein SR, Koziol-McLain J, Barta DC, Steiner J. Chart reviews in emergency medicine research: Where are the methods?. Ann Emerg Med. 1996;27(3):305–8. doi: 10.1016/s0196-0644(96)70264-0 8599488

[20] Bisdorff A, Von Brevern M, Lempert T, Newman-Toker DE. Classification of vestibular symptoms: towards an international classification of vestibular disorders. J Vestib Res. 2009;19(1–2):1–13. doi: 10.3233/VES-2009-0343 19893191

[21] Newman-Toker DE, Edlow JA. TiTrATE: A Novel, Evidence-Based Approach to Diagnosing Acute Dizziness and Vertigo. Neurol Clin. 2015;33(3):577–99, viii. doi: 10.1016/j.ncl.2015.04.011 26231273 PMC4522574

[22] Johnston SC, Rothwell PM, Nguyen-Huynh MN, Giles MF, Elkins JS, Bernstein AL, et al. Validation and refinement of scores to predict very early stroke risk after transient ischaemic attack. Lancet. 2007;369(9558):283–92. doi: 10.1016/S0140-6736(07)60150-0 17258668

[23] Palm F, Urbanek C, Wolf J, Buggle F, Kleemann T, Hennerici MG, et al. Etiology, risk factors and sex differences in ischemic stroke in the Ludwigshafen Stroke Study, a population-based stroke registry. Cerebrovasc Dis. 2012;33(1):69–75. doi: 10.1159/000333417 22133999

[24] Bir S, Kelley RE. Antithrombotic Therapy in the Prevention of Stroke. Biomedicines. 2021;9(12):1906. doi: 10.3390/biomedicines9121906 34944719 PMC8698439

[25] Bushnell C, Kernan WN, Sharrief AZ, Chaturvedi S, Cole JW, Cornwell WK 3rd, et al. 2024 Guideline for the Primary Prevention of Stroke: A Guideline From the American Heart Association/American Stroke Association. Stroke. 2024;55(12):e344–424. doi: 10.1161/STR.0000000000000475 39429201

[26] Mulder MJHL, Cras TY, Shay J, Dippel DWJ, Burke JF. Comparison of American and European Guideline Recommendations for Diagnostic Workup and Secondary Prevention of Ischemic Stroke and Transient Ischemic Attack. Circulation. 2024;150(10):806–15. doi: 10.1161/CIRCULATIONAHA.124.069651 39226381

[27] Diener H-C, Bogousslavsky J, Brass LM, Cimminiello C, Csiba L, Kaste M, et al. Aspirin and clopidogrel compared with clopidogrel alone after recent ischaemic stroke or transient ischaemic attack in high-risk patients (MATCH): randomised, double-blind, placebo-controlled trial. Lancet. 2004;364(9431):331–7. doi: 10.1016/S0140-6736(04)16721-4 15276392

[28] Bhatt DL, Fox KAA, Hacke W, Berger PB, Black HR, Boden WE, et al. Clopidogrel and aspirin versus aspirin alone for the prevention of atherothrombotic events. N Engl J Med. 2006;354(16):1706–17. doi: 10.1056/NEJMoa060989 16531616

[29] SPS3 Investigators, Benavente OR, Hart RG, McClure LA, Szychowski JM, Coffey CS, et al. Effects of clopidogrel added to aspirin in patients with recent lacunar stroke. N Engl J Med. 2012;367(9):817–25. doi: 10.1056/NEJMoa1204133 22931315 PMC4067036

[30] Levine GN, Bates ER, Bittl JA, Brindis RG, Fihn SD, Fleisher LA, et al. 2016 ACC/AHA Guideline Focused Update on Duration of Dual Antiplatelet Therapy in Patients With Coronary Artery Disease: A Report of the American College of Cardiology/American Heart Association Task Force on Clinical Practice Guidelines: An Update of the 2011 ACCF/AHA/SCAI Guideline for Percutaneous Coronary Intervention, 2011 ACCF/AHA Guideline for Coronary Artery Bypass Graft Surgery, 2012 ACC/AHA/ACP/AATS/PCNA/SCAI/STS Guideline for the Diagnosis and Management of Patients With Stable Ischemic Heart Disease, 2013 ACCF/AHA Guideline for the Management of ST-Elevation Myocardial Infarction, 2014 AHA/ACC Guideline for the Management of Patients With Non-ST-Elevation Acute Coronary Syndromes, and 2014 ACC/AHA Guideline on Perioperative Cardiovascular Evaluation and Management of Patients Undergoing Noncardiac Surgery. Circulation. 2016;134(10):e123-55. doi: 10.1161/cir.0000000000000404 27026020

[31] Levine GN, Bates ER, Bittl JA, Brindis RG, Fihn SD, Fleisher LA, et al. 2016 ACC/AHA Guideline Focused Update on Duration of Dual Antiplatelet Therapy in Patients With Coronary Artery Disease: A Report of the American College of Cardiology/American Heart Association Task Force on Clinical Practice Guidelines. J Am Coll Cardiol. 2016;68(10):1082–115. doi: 10.1016/j.jacc.2016.03.513 27036918

[32] Valgimigli M, Bueno H, Byrne RA, Collet J-P, Costa F, Jeppsson A, et al. 2017 ESC focused update on dual antiplatelet therapy in coronary artery disease developed in collaboration with EACTS: The Task Force for dual antiplatelet therapy in coronary artery disease of the European Society of Cardiology (ESC) and of the European Association for Cardio-Thoracic Surgery (EACTS). Eur Heart J. 2018;39(3):213–60. doi: 10.1093/eurheartj/ehx419 28886622

[33] Cheung CSK, Mak PSK, Manley KV, Lam JMY, Tsang AYL, Chan HMS, et al. Predictors of important neurological causes of dizziness among patients presenting to the emergency department. Emerg Med J. 2010;27(7):517–21. doi: 10.1136/emj.2009.078014 20584952

[34] Hanna J, Malhotra A, Brauer PR, Luryi A, Michaelides E. A comparison of benign positional vertigo and stroke patients presenting to the emergency department with vertigo or dizziness. Am J Otolaryngol. 2019;40(6):102263. doi: 10.1016/j.amjoto.2019.07.007 31358317

[35] Flaherty ML. Anticoagulant-associated intracerebral hemorrhage. Semin Neurol. 2010;30(5):565–72. doi: 10.1055/s-0030-1268866 21207349

[36] Ha ACT, Bhatt DL, Rutka JT, Johnston SC, Mazer CD, Verma S. Intracranial hemorrhage during dual antiplatelet therapy: JACC review topic of the week. J Am Coll Cardiol. 2021;78(13):1372–84. doi: 10.1016/j.jacc.2021.07.048 34556323

[37] Lanas-Gimeno A, Lanas A. Risk of gastrointestinal bleeding during anticoagulant treatment. Expert Opin Drug Saf. 2017;16(6):673–85. doi: 10.1080/14740338.2017.1325870 28467190

[38] Tan VP, Yan BP, Kiernan TJ, Ajani AE. Risk and management of upper gastrointestinal bleeding associated with prolonged dual-antiplatelet therapy after percutaneous coronary intervention. Cardiovasc Revasc Med. 2009;10(1):36–44. doi: 10.1016/j.carrev.2008.11.001 19159853

[39] Neto ACL, Kim J-S, Bernardo WM, Bittar RSM. Vertigo and dizziness due to vertebrobasilar TIA: a prospective study. Front Stroke. 2024;3:1429068. doi: 10.3389/fstro.2024.1429068 41542262 PMC12802699

[40] Savitz SI, Caplan LR, Edlow JA. Pitfalls in the diagnosis of cerebellar infarction. Acad Emerg Med. 2007;14(1):63–8. doi: 10.1197/j.aem.2006.06.060 17200515

[41] Fischer U, Baumgartner A, Arnold M, Nedeltchev K, Gralla J, De Marchis GM, et al. What is a minor stroke?. Stroke. 2010;41(4):661–6. doi: 10.1161/STROKEAHA.109.572883 20185781

[42] Khatri P, Kleindorfer DO, Yeatts SD, Saver JL, Levine SR, Lyden PD, et al. Strokes with minor symptoms: an exploratory analysis of the National Institute of Neurological Disorders and Stroke recombinant tissue plasminogen activator trials. Stroke. 2010;41(11):2581–6. doi: 10.1161/STROKEAHA.110.593632 20814000 PMC2964419

[43] Lavallée P, Amarenco P. TIA clinic: a major advance in management of transient ischemic attacks. Front Neurol Neurosci. 2014;33:30–40. doi: 10.1159/000351890 24157555

[44] Luengo-Fernandez R, Gray AM, Rothwell PM. Effect of urgent treatment for transient ischaemic attack and minor stroke on disability and hospital costs (EXPRESS study): a prospective population-based sequential comparison. Lancet Neurol. 2009;8(3):235–43. doi: 10.1016/S1474-4422(09)70019-5 19200786

[45] Ryan DJ, Kenny RA, Christensen S, Meaney JFM, Fagan AJ, Harbison J. Ischaemic stroke or TIA in older subjects associated with impaired dynamic blood pressure control in the absence of severe large artery stenosis. Age Ageing. 2015;44(4):655–61. doi: 10.1093/ageing/afv011 25716898

