# Supplementary figures and images for: Association of anticoagulant and antiplatelet therapy with acute cerebral infarction in patients presenting with isolated vertigo or dizziness: A retrospective cohort study
Source: PLoS One. 2026 Jun 11;21(6):e0350671. doi: 10.1371/journal.pone.0350671 (PMC13258147; doi:10.1371/journal.pone.0350671)

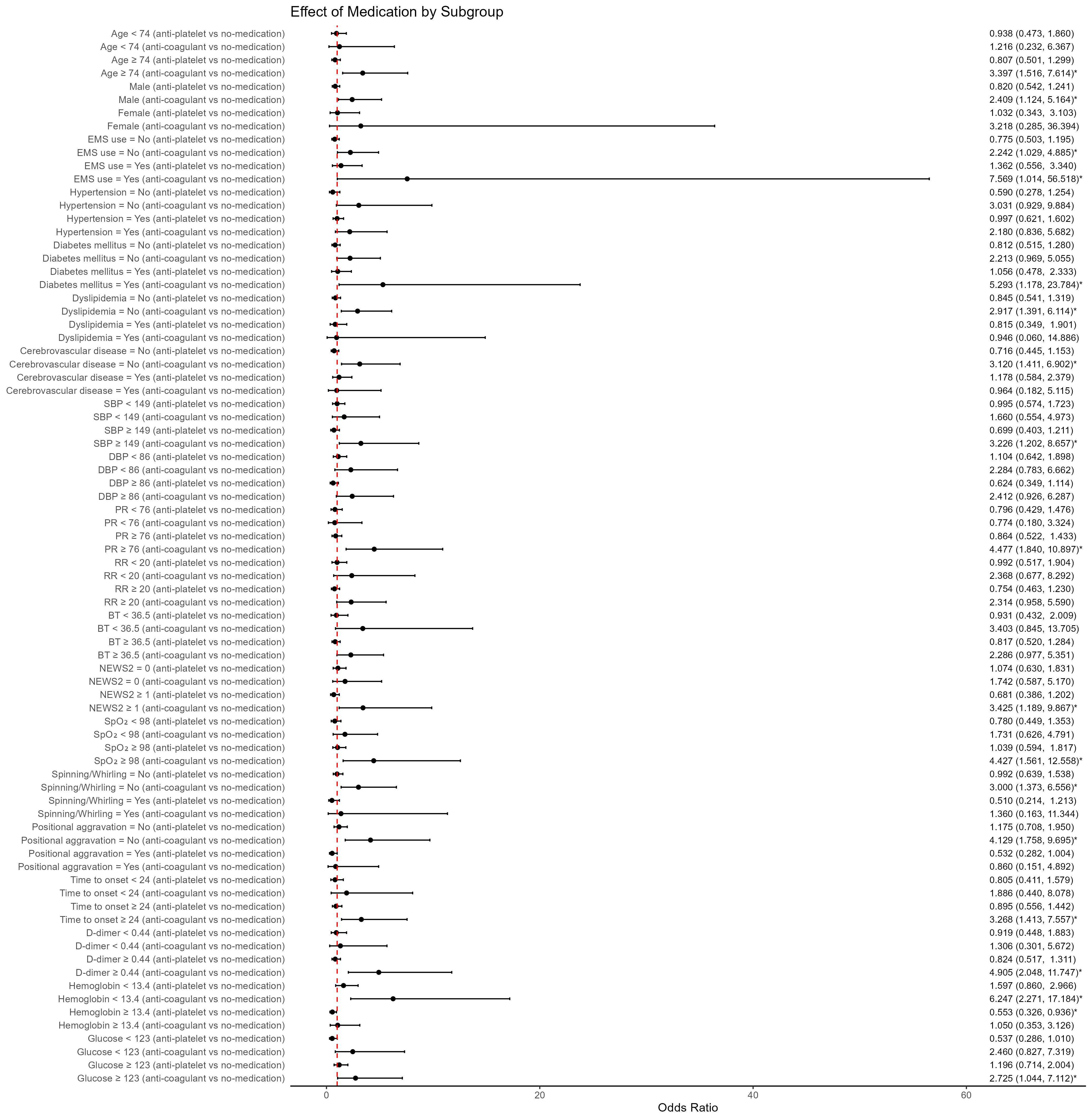

Supplement: S1 Fig — Covariates included in multivariable model B were used for adjustment, except for the stratifying variable in each subgroup analysis. (TIF) [file pone.0350671.s001.tif]

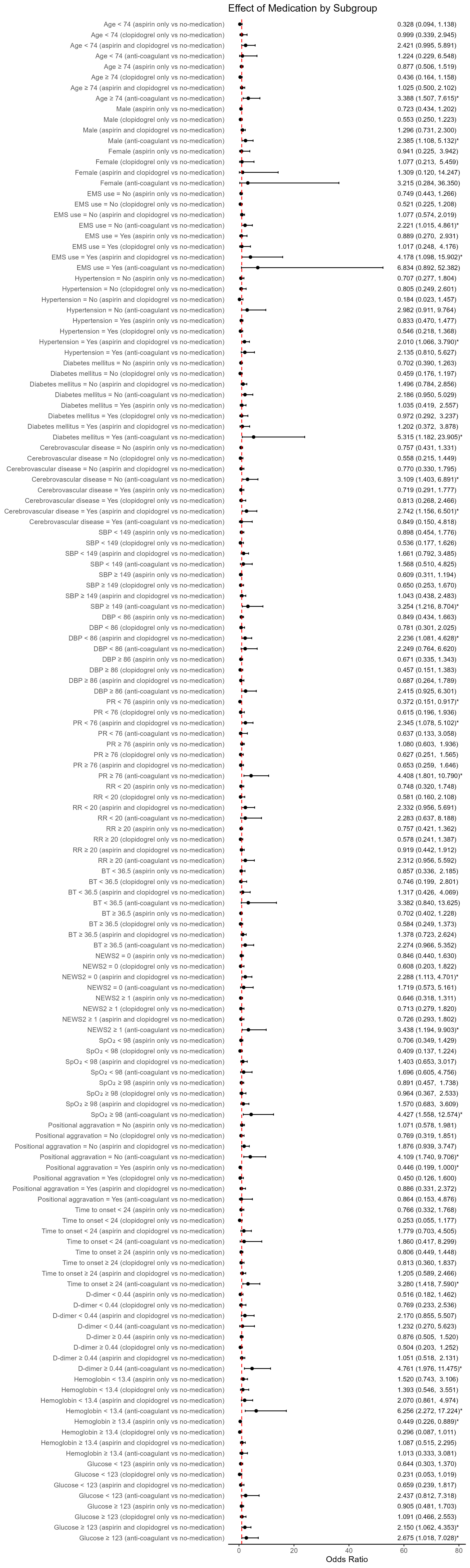

Supplement: S2 Fig — Covariates included in multivariable model B were used for adjustment, except for the stratifying variable in each subgroup analysis. (TIF) [file pone.0350671.s002.tif]

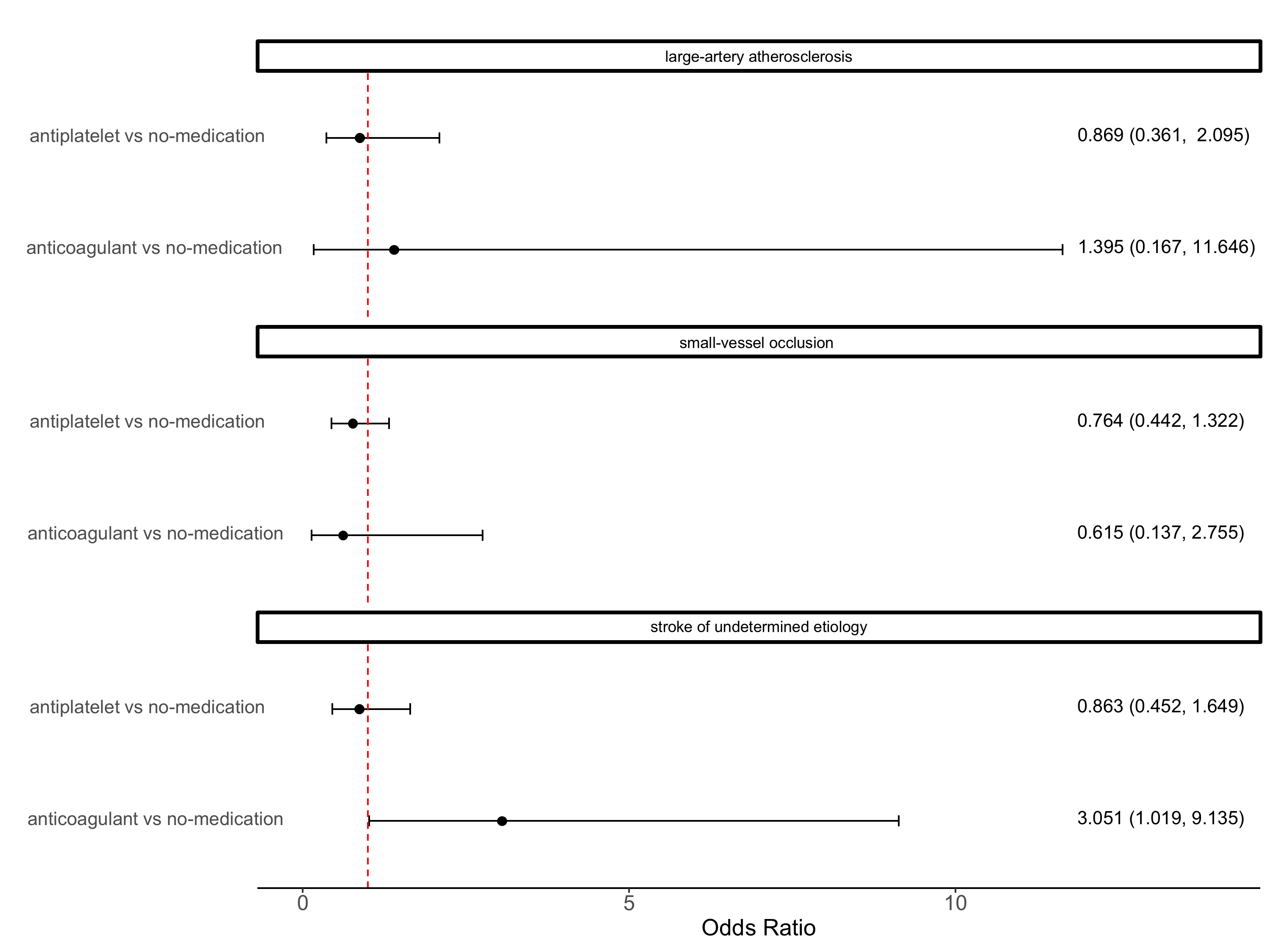

Supplement: S3 Fig — Forest plot showing the adjusted odds ratios for the association between medication group and each TOAST-defined subtype of ACI. Separate logistic regression models were fitted for each subtype, adjusting for covariates in model B. Patients with other subtypes were treated as missing. (TIF) [file pone.0350671.s003.tif]

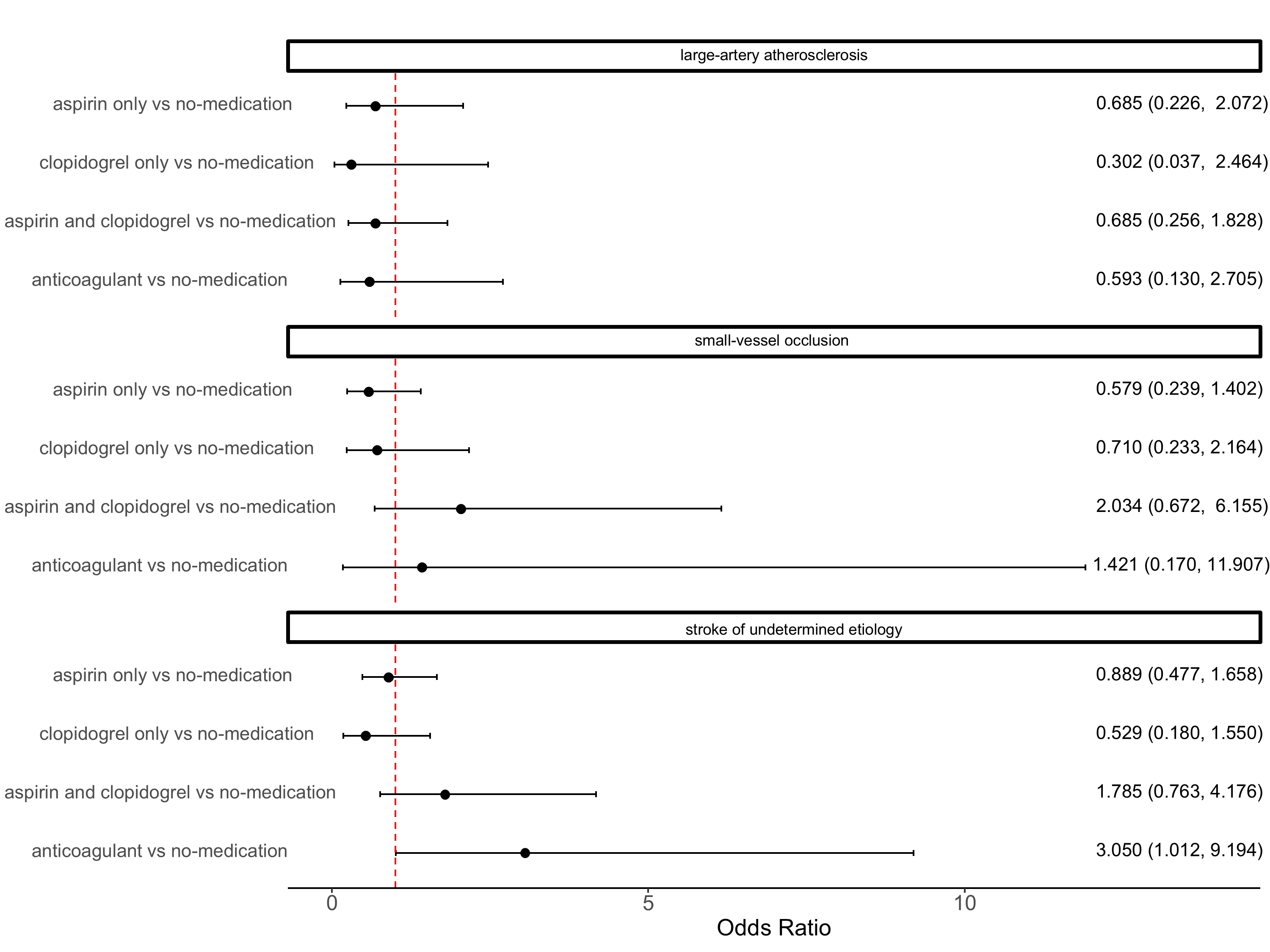

Supplement: S4 Fig — Forest plot showing the adjusted odds ratios for the association between medication group and each TOAST-defined subtype of ACI. Separate logistic regression models were fitted for each subtype, adjusting for covariates in model B. Patients with other subtypes were treated as missing. (TIF) [file pone.0350671.s004.tif]
