# Supplementary material for: Association of anticoagulant and antiplatelet therapy with acute cerebral infarction in patients presenting with isolated vertigo or dizziness: A retrospective cohort study
Source: PLoS One. 2026 Jun 11;21(6):e0350671. doi: 10.1371/journal.pone.0350671 (PMC13258147; doi:10.1371/journal.pone.0350671)
Supplement: S1 Table — (DOCX) [file pone.0350671.s005.docx]

**S1 Table. Comparison among no-medication, antiplatelet, anticoagulant group.**

| **Variable** | **No-medication (n=1104)** | **Antiplatelet (n=670)** | **Anticoagulant (n=101)** | **P value** |
| --- | --- | --- | --- | --- |
| Age, year | 74.0 [69.0;78.0] | 75.0 [73.0;80.0] | 75.0 [73.0;80.0] | <0.001 |
| **Age groups, n (%)** |  |  |  | <0.001 |
| <65 | 176 (15.9) | 25 (3.7) | 2 (2.0) |  |
| 65-74 | 441 (39.9) | 258 (38.5) | 46 (45.5) |  |
| ≥75 | 487 (44.1) | 387 (57.8) | 53 (52.5) |  |
| Male, n (%) | 696 (63.0) | 563 (84.0) | 83 (82.2) | <0.001 |
| EMS use, n (%) | 178 (16.1) | 108 (16.1) | 23 (22.8) | 0.215 |
| **Comorbidity, n (%)** |  |  |  |  |
| Hypertension | 627 (56.8) | 505 (75.4) | 68 (67.3) | <0.001 |
| Diabetes mellitus | 170 (15.4) | 208 (31.0) | 19 (18.8) | <0.001 |
| Dyslipidemia | 129 (11.7) | 129 (19.3) | 19 (18.8) | <0.001 |
| Cerebrovascular disease | 114 (10.3) | 186 (27.8) | 23 (22.8) | <0.001 |
| Chronic kidney disease | 31 (2.8) | 61 (9.1) | 7 (6.9) | <0.001 |
| Coronary artery disease | 58 (5.3) | 200 (29.9) | 12 (11.9) | <0.001 |
| Atrial fibrillation | 6 (0.5) | 13 (1.9) | 51 (50.5) | <0.001 |
| COPD / Asthma | 33 (3.0) | 34 (5.1) | 5 (5.0) | 0.072 |
| **Physiology** |  |  |  |  |
| SBP, mmHg | 149.0 [132.0;168.0] | 149.0 [129.0;169.0] | 150.0 [129.0;166.0] | 0.878 |
| DBP, mmHg | 87.0 [78.0;96.0] | 83.0 [74.0;94.0] | 86.0 [77.0;96.0] | <0.001 |
| PR, bpm | 78.0 [68.0;87.0] | 76.0 [66.0;85.0] | 75.0 [64.0;86.0] | 0.014 |
| RR, bpm | 20.0 [18.0;20.0] | 20.0 [18.0;20.0] | 20.0 [18.0;20.0] | 0.653 |
| BT, ˚C | 36.5 [36.5;36.6] | 36.5 [36.4;36.6] | 36.5 [36.4;36.6] | 0.422 |
| NEWS2 | 0.0 [0.0;1.0] | 0.0 [0.0;1.0] | 0.0 [ 0.0; 1.0] | 0.371 |
| SpO₂, % | 98.0 [96.0;98.0] | 98.0 [96.0;98.0] | 98.0 [96.0;98.0] | 0.658 |
| Admission, n (%) | 72 (6.5) | 74 (11.0) | 18 (17.8) | <0.001 |
| **Dizziness feature, n (%)** |  |  |  |  |
| Spinning/Whirling | 376 (34.1) | 191 (28.5) | 22 (21.8) | 0.005 |
| Positional vertigo/dizziness | 566 (51.3) | 307 (45.8) | 34 (33.7) | 0.001 |
| Any nystagmus | 49 (4.4) | 23 (3.4) | 1 (1.0) | 0.171 |
| Continuous (vs episodic) dizziness | 723 (65.5) | 450 (67.2) | 81 (80.2) | 0.011 |
| **Duration of symptom, n (%)** |  |  |  | 0.055 |
| <10 min | 349 (31.6) | 206 (30.7) | 18 (17.8) |  |
| 10-59 min | 21 (1.9) | 11 (1.6) | 1 (1.0) |  |
| ≥60 min | 734 (66.5) | 453 (67.6) | 82 (81.2) |  |
| Time to onset, hr | 24.0 [5.0;120.0] | 24.0 [5.0;168.0] | 24.0 [5.0;72.0] | 0.565 |
| D-dimer, mg/L (n=1846) | 0.4 [0.3;0.8] | 0.5 [0.3;0.9] | 0.3 [0.2; 0.7] | <0.001 |
| Hemoglobin | 13.4 [12.4;14.5] | 13.3 [12.2;14.5] | 13.4 [12.5;14.2] | 0.408 |
| Glucose | 120.5 [106.0;141.5] | 129.0 [111.0;154.0] | 126.0 [108.0;145.0] | <0.001 |
| **TOAST classification (n=153), n (%)** |  |  |  | <0.001 |
| Large artery atherosclerosis | 12 (16.2) | 12 (20.3) | 1 (5.0) |  |
| Cardioembolism | 0 (0.0) | 1 (1.7) | 7 (35.0) |  |
| Small-vessel occlusion | 38 (51.4) | 25 (42.4) | 3 (15.0) |  |
| Stroke of other determined etiology | 0 (0.0) | 1 (1.7) | 0 (0.0) |  |
| Stroke of undetermined etiology | 24 (32.4) | 20 (33.9) | 9 (45.0) |  |
| **Medication, n (%)** |  |  |  |  |
| Aspirin | 0 (0.0) | 518 (77.3) | 13 (12.9) | <0.001 |
| Clopidogrel | 0 (0.0) | 293 (43.7) | 5 (5.0) | <0.001 |
| Aspirin or Clopidogrel | 0 (0.0) | 670 (100.0) | 17 (16.8) | <0.001 |
| Warfarin | 0 (0.0) | 0 (0.0) | 12 (11.9) | <0.001 |
| Non-vitamin K oral anticoagulant (NOAC) | 0 (0.0) | 0 (0.0) | 89 (88.1) | <0.001 |
| Warfarin or NOAC | 0 (0.0) | 0 (0.0) | 101 (100.0) | <0.001 |
| **Five groups, n (%)** |  |  |  | <0.001 |
| No-medication | 1104 (100.0) | 0 (0.0) | 0 (0.0) |  |
| Aspirin | 0 (0.0) | 377 (56.3) | 0 (0.0) |  |
| Clopidogrel | 0 (0.0) | 152 (22.7) | 0 (0.0) |  |
| Aspirin and Clopidogrel | 0 (0.0) | 141 (21.0) | 0 (0.0) |  |
| Anticoagulant | 0 (0.0) | 0 (0.0) | 101 (100.0) |  |

Abbreviation. EMS, emergency medical service; COPD, chronic obstructive pulmonary disease; SBP, systolic blood pressure; DBP, diastolic blood pressure; PR, pulse rate; RR, respiratory rate; BT, body temperature; NEWS2, national early warning score 2; SpO2, peripheral capillary oxygen saturation; ICU, intensive care unit; min, minute; hr, hour; mg/L, milligrams per liter; TOAST, trial of ORG 10172 in acute stroke treatment. Continuous variables are presented as median [IQR].
