# Supplementary material for: Association of anticoagulant and antiplatelet therapy with acute cerebral infarction in patients presenting with isolated vertigo or dizziness: A retrospective cohort study
Source: PLoS One. 2026 Jun 11;21(6):e0350671. doi: 10.1371/journal.pone.0350671 (PMC13258147; doi:10.1371/journal.pone.0350671)
Supplement: S2 Table — (DOCX) [file pone.0350671.s006.docx]

**S2 Table. Comparison among no-medication, aspirin-only group, clopidogrel-only group, aspirin and Clopidogrel group, and anti-coagulant group.**

| **Variable** | **No-medication (n=1104)** | **Aspirin-only (n=377)** | **Clopidogrel-only (n=152)** | **Aspirin and Clopidogrel (n=141)** | **Anticoagulant (n=101)** | **P value** |
| --- | --- | --- | --- | --- | --- | --- |
| Age, year | 74.0 [69.0;78.0] | 75.0 [73.0;80.0] | 75.0 [73.0;81.5] | 75.0 [72.0;78.0] | 75.0 [73.0;80.0] | <0.001 |
| **Age groups, n (%)** |  |  |  |  |  | <0.001 |
| <65 | 176 (15.9) | 13 (3.4) | 7 (4.6) | 5 (3.5) | 2 (2.0) |  |
| 65-74 | 441 (39.9) | 140 (37.1) | 60 (39.5) | 58 (41.1) | 46 (45.5) |  |
| ≥75 | 487 (44.1) | 224 (59.4) | 85 (55.9) | 78 (55.3) | 53 (52.5) |  |
| Male, n (%) | 696 (63.0) | 314 (83.3) | 114 (75.0) | 135 (95.7) | 83 (82.2) | <0.001 |
| EMS use, n (%) | 178 (16.1) | 59 (15.6) | 34 (22.4) | 15 (10.6) | 23 (22.8) | 0.033 |
| **Comorbidity, n (%)** |  |  |  |  |  |  |
| Hypertension | 627 (56.8) | 285 (75.6) | 112 (73.7) | 108 (76.6) | 68 (67.3) | <0.001 |
| Diabetes mellitus | 170 (15.4) | 121 (32.1) | 44 (28.9) | 43 (30.5) | 19 (18.8) | <0.001 |
| Dyslipidemia | 129 (11.7) | 74 (19.6) | 28 (18.4) | 27 (19.1) | 19 (18.8) | <0.001 |
| Cerebrovascular disease | 114 (10.3) | 92 (24.4) | 44 (28.9) | 50 (35.5) | 23 (22.8) | <0.001 |
| Chronic kidney disease | 31 (2.8) | 35 (9.3) | 15 (9.9) | 11 (7.8) | 7 (6.9) | <0.001 |
| Coronary artery disease | 58 (5.3) | 89 (23.6) | 47 (30.9) | 64 (45.4) | 12 (11.9) | <0.001 |
| Atrial fibrillation | 6 (0.5) | 5 (1.3) | 6 (3.9) | 2 (1.4) | 51 (50.5) | <0.001 |
| COPD / Asthma | 33 (3.0) | 17 (4.5) | 9 (5.9) | 8 (5.7) | 5 (5.0) | 0.197 |
| **Physiology** |  |  |  |  |  |  |
| SBP, mmHg | 149.0 [132.0;168.0] | 151.0 [129.0;171.0] | 151.5 [133.0;171.0] | 144.0 [129.0;163.0] | 150.0 [129.0;166.0] | 0.132 |
| DBP, mmHg | 87.0 [78.0;96.0] | 83.0 [74.0;94.0] | 83.5 [73.5;96.5] | 83.0 [74.0;94.0] | 86.0 [77.0;96.0] | <0.001 |
| PR, bpm | 78.0 [68.0;87.0] | 75.0 [65.0;84.0] | 77.0 [67.0;88.0] | 75.0 [66.0;84.0] | 75.0 [64.0;86.0] | 0.043 |
| RR, bpm | 20.0 [18.0;20.0] | 20.0 [18.0;20.0] | 20.0 [18.0;20.0] | 20.0 [18.0;20.0] | 20.0 [18.0;20.0] | 0.892 |
| BT, ˚C | 36.5 [36.5;36.6] | 36.5 [36.4;36.6] | 36.5 [36.4;36.6] | 36.5 [36.4;36.6] | 36.5 [36.4;36.6] | 0.685 |
| NEWS2 | 0.0 [0.0;1.0] | 0.0 [0.0;1.0] | 0.0 [0.0;1.0] | 0.0 [0.0;1.0] | 0.0 [0.0;1.0] | 0.082 |
| SpO₂, % | 98.0 [96.0;98.0] | 98.0 [96.0;98.0] | 97.0 [96.0;98.0] | 97.0 [96.0;98.0] | 98.0 [96.0;98.0] | 0.413 |
| Admission, n (%) | 72 (6.5) | 37 (9.8) | 15 (9.9) | 22 (15.6) | 18 (17.8) | <0.001 |
| **Dizziness feature, n (%)** |  |  |  |  |  |  |
| Spinning/Whirling | 376 (34.1) | 113 (30.0) | 47 (30.9) | 31 (22.0) | 22 (21.8) | 0.007 |
| Positional vertigo/dizziness | 566 (51.3) | 189 (50.1) | 64 (42.1) | 54 (38.3) | 34 (33.7) | <0.001 |
| Any nystagmus | 49 (4.4) | 12 (3.2) | 6 (3.9) | 5 (3.5) | 1 (1.0) | 0.447 |
| Continuous (vs episodic) dizziness | 723 (65.5) | 237 (62.9) | 105 (69.1) | 108 (76.6) | 81 (80.2) | 0.001 |
| **Duration of symptom, n (%)** |  |  |  |  |  | 0.012 |
| <10 min | 349 (31.6) | 133 (35.3) | 42 (27.6) | 31 (22.0) | 18 (17.8) |  |
| 10-59 min | 21 (1.9) | 5 (1.3) | 4 (2.6) | 2 (1.4) | 1 (1.0) |  |
| ≥60 min | 734 (66.5) | 239 (63.4) | 106 (69.7) | 108 (76.6) | 82 (81.2) |  |
| Time to onset, hr | 24.0 [5.0;120.0] | 24.0 [5.0;120.0] | 48.0 [4.0;168.0] | 24.0 [5.0;120.0] | 24.0 [5.0;72.0] | 0.845 |
| D-dimer, mg/L (n=1846) | 0.4 [0.3;0.8] | 0.5 [0.3;0.9] | 0.6 [0.3;1.0] | 0.5 [0.3;1.0] | 0.3 [0.2;0.7] | <0.001 |
| Hemoglobin | 13.4 [12.4;14.5] | 13.3 [12.3;14.4] | 13.2 [12.1;14.5] | 13.3 [12.1;14.8] | 13.4 [12.5;14.2] | 0.720 |
| Glucose | 120.5 [106.0;141.5] | 129.0 [111.0;155.0] | 129.5 [111.5;150.5] | 127.0 [109.0;155.0] | 126.0 [108.0;145.0] | <0.001 |
| **TOAST classification (n=153), n (%)** |  |  |  |  |  | <0.001 |
| Large artery atherosclerosis | 12 (16.2) | 5 (17.9) | 1 (10.0) | 6 (28.6) | 1 (5.0) |  |
| Cardioembolism | 0 (0.0) | 0 (0.0) | 1 (10.0) | 0 (0.0) | 7 (35.0) |  |
| Small-vessel occlusion | 38 (51.4) | 16 (57.1) | 4 (40.0) | 5 (23.8) | 3 (15.0) |  |
| Stroke of other determined etiology | 0 (0.0) | 0 (0.0) | 0 (0.0) | 1 (4.8) | 0 (0.0) |  |
| Stroke of undetermined etiology | 24 (32.4) | 7 (25.0) | 4 (40.0) | 9 (42.9) | 9 (45.0) |  |
| **Medication, n (%)** |  |  |  |  |  |  |
| Aspirin | 0 (0.0) | 377 (100.0) | 0 (0.0) | 141 (100.0) | 13 (12.9) | <0.001 |
| Clopidogrel | 0 (0.0) | 0 (0.0) | 152 (100.0) | 141 (100.0) | 5 (5.0) | <0.001 |
| Aspirin or Clopidogrel | 0 (0.0) | 377 (100.0) | 152 (100.0) | 141 (100.0) | 17 (16.8) | <0.001 |
| Warfarin | 0 (0.0) | 0 (0.0) | 0 (0.0) | 0 (0.0) | 12 (11.9) | <0.001 |
| Non-vitamin K oral anticoagulant (NOAC) | 0 (0.0) | 0 (0.0) | 0 (0.0) | 0 (0.0) | 89 (88.1) | <0.001 |
| Warfarin or NOAC | 0 (0.0) | 0 (0.0) | 0 (0.0) | 0 (0.0) | 101 (100.0) | <0.001 |
| **Three groups, n (%)** |  |  |  |  |  | <0.001 |
| No-medication | 1104 (100.0) | 0 (0.0) | 0 (0.0) | 0 (0.0) | 0 (0.0) |  |
| Antiplatelet | 0 (0.0) | 377 (100.0) | 152 (100.0) | 141 (100.0) | 0 (0.0) |  |
| Anticoagulant | 0 (0.0) | 0 (0.0) | 0 (0.0) | 0 (0.0) | 101 (100.0) |  |

Abbreviation. EMS, emergency medical service; COPD, chronic obstructive pulmonary disease; SBP, systolic blood pressure; DBP, diastolic blood pressure; PR, pulse rate; RR, respiratory rate; BT, body temperature; NEWS2, national early warning score 2; SpO2, peripheral capillary oxygen saturation; ICU, intensive care unit; min, minute; hr, hour; mg/L, milligrams per liter; TOAST, trial of ORG 10172 in acute stroke treatment. Continuous variables are presented as median [IQR].
