# Supplementary material for: Association of anticoagulant and antiplatelet therapy with acute cerebral infarction in patients presenting with isolated vertigo or dizziness: A retrospective cohort study
Source: PLoS One. 2026 Jun 11;21(6):e0350671. doi: 10.1371/journal.pone.0350671 (PMC13258147; doi:10.1371/journal.pone.0350671)
Supplement: S1 File — (DOCX) [file pone.0350671.s007.docx]

STROBE Checklist for Observational Study

| Item No. | Checklist Item | Your Study (Response) |
| --- | --- | --- |
| 1 | Title and abstract | The study is clearly identified as a retrospective cohort study in both the title and the abstract. The abstract includes a structured summary of background, objectives, study design, setting, participants, key findings (including incidence of infarction and antithrombotic agent association), and conclusions. |
| 2 | Background/rationale | The study aims to evaluate the incidence of ACI among ED patients with IVD and the association with antiplatelet or anticoagulant use. |
| 3 | Objectives | To determine the incidence of ACI in IVD patients and evaluate the effect of antithrombotic medications. |
| 4 | Study design | Retrospective cohort study. |
| 5 | Setting | Conducted at the emergency department of a tertiary care center in Seoul, Korea, over a multi-year period (2019–2022). |
| 6 | Participants | Included all adult ED patients presenting with IVD who underwent DWI MRI. Exclusion criteria: altered mental status, trauma, or known secondary causes. |
| 7 | Variables | Outcome: ACI identified on DWI. Exposure: use of antiplatelet or anticoagulant medications. Covariates include age, comorbidities, vital signs |
| 8 | Data sources/measurement | EMR review for demographics, medication use, imaging. DWI confirmed by radiologist. |
| 9 | Bias | Selection bias due to only including patients who underwent brain MRI. |
| 10 | Study size | Total of 1,875 patients included after applying inclusion/exclusion criteria. |
| 11 | Quantitative variables | Logistic regression used to calculate ORs and 95% CI for ACI incidence. |
| 12 | Statistical methods | Univariate and multivariate logistic regression; subgroup and sensitivity analyses also performed. |
| 13 | Participants | Patient flow and selection described in the text; flowchart included. |
| 14 | Descriptive data | Table 1 provides demographics, vital signs, medication usage. |
| 15 | Outcome data | Table 2 shows AUC curve, sensitivity, specificity, diagnostic accuracy, positive predictive value, negative predictive value, positive likelihood ratio, negative likelihood ratio of each subgroup of the study. |
| 16 | Main results | Nearly 20% of patients in the anticoagulant group and 15% in the dual antiplatelet group (aspirin and clopidogrel) experienced ACI among ED patients with IVD. |
| 17 | Other analyses | Subgroup analysis by medication type and stroke subtype; sensitivity analysis using TOAST classification. |
| 18 | Key results | anticoagulant use was significantly associated with incidence of ACI in IVD patients. |
| 19 | Limitations | Selection bias due to MRI-based inclusion; retrospective design limits causal inference. |
| 20 | Interpretation | The use of anticoagulants was a significant predictor of ACI occurrence; however, more prospective studies are needed. |
| 21 | Generalisability | Single-center study may limit generalizability to broader populations. |
| 22 | Funding | This research was supported by a grant (VHSMC25012) from VHS Veterans Medical Research Institute. |
| 23 | Conflicts of interest | All authors declare no conflicts of interest. |
| 24 | Ethical approval | IRB approval obtained; IRB No. 2025-03-013 |
| 25 | Data availability | Datasets available from corresponding author upon reasonable request. |
